# Geppetto: a reusable modular open platform for exploring neuroscience data and models

**DOI:** 10.1098/rstb.2017.0380

**Published:** 2018-09-10

**Authors:** Matteo Cantarelli, Boris Marin, Adrian Quintana, Matt Earnshaw, Robert Court, Padraig Gleeson, Salvador Dura-Bernal, R. Angus Silver, Giovanni Idili

**Affiliations:** 1 OpenWorm Foundation, USA; 2 MetaCell Limited, UK; 3 Department of Neuroscience, Physiology and Pharmacology, University College London, UK; 4 Departamento de Física, Faculdade de Filosofia, Ciências e Letras de Ribeirão Preto, Universidade de São Paulo, Brazil; 5 EyeSeeTea Limited, UK; 6 Institute for Adaptive and Neural Computation, School of Informatics, University of Edinburgh, Edinburgh, UK; 7 Department of Physiology and Pharmacology, SUNY Downstate, Brooklyn, NY, USA

**Keywords:** computational neuroscience, neuroinformatics, computational biology, modelling and simulation, scientific software, data visualization

## Abstract

Geppetto is an open-source platform that provides generic middleware infrastructure for building both online and desktop tools for visualizing neuroscience models and data and managing simulations. Geppetto underpins a number of neuroscience applications, including Open Source Brain (OSB), Virtual Fly Brain (VFB), NEURON-UI and NetPyNE-UI. OSB is used by researchers to create and visualize computational neuroscience models described in NeuroML and simulate them through the browser. VFB is the reference hub for *Drosophila melanogaster* neural anatomy and imaging data including neuropil, segmented neurons, microscopy stacks and gene expression pattern data. Geppetto is also being used to build a new user interface for NEURON, a widely used neuronal simulation environment, and for NetPyNE, a Python package for network modelling using NEURON. Geppetto defines domain agnostic abstractions used by all these applications to represent their models and data and offers a set of modules and components to integrate, visualize and control simulations in a highly accessible way. The platform comprises a backend which can connect to external data sources, model repositories and simulators together with a highly customizable frontend.

This article is part of a discussion meeting issue ‘Connectome to behaviour: modelling *C. elegans* at cellular resolution’.

## 1. Introduction

Investigations of fundamental questions in neuroscience, such as the mechanistic basis of behaviour and cognition, generate large volumes of experimental data as well as complex computational models spanning different levels of biological detail. These push the neuroscience applications available to researchers to their limits. Visualizing and managing the heterogeneity of neuroscience data and models in a way that is accessible and usable for both experimentalists and modellers is crucial for driving the field forward. For example, it has been challenging to visualize the data and models required to link the dynamics of the nervous system of *Caenorhabditis elegans* to its behaviour [1], or to understand how the sleep regulatory circuit in *Drosophila melanogaster* is affected by the surrounding environment [2].

In neuroscience, visualization and simulation tools exist for many of the levels of detail involved [3–7], but it is often far from trivial to use them in concert [8]. One popular approach to solving this issue involves using general purpose programming languages such as Python [9–11]. This approach enables the rapid development of toolchains to solve a specific visualization and integration problem, gluing together multiple libraries and tools [12]. The problem with this approach is that these toolchains are usually developed for a specific use case, e.g. processing data from a specific source. Over time, as the application is modified to solve different problems (e.g. deal with a new model or with a new type of visualization), the specificity becomes an obstacle and the codebase becomes a series of ad hoc extensions that are difficult to maintain [13]. An even greater problem comes from the fact that these tools, and even more so their combination, are rather inaccessible to many researchers. Such technological barriers have had a remarkable effect in the neuroscience field as a whole, resulting in modellers and experimentalists working as two different communities separated by a technological divide. This has resulted in computational models that are poorly validated and has left model-generated hypotheses unexplored.

Data and models come in many different types, which are subject to change as the field evolves. Handling such heterogeneities constitutes a significant challenge for neuroscience applications, given that not all of the formats that will be required to answer novel scientific questions will be known at design time. Standard neuroscience formats that have emerged to date include NeuroML [14,15] for computational neuroscience and Neurodata Without Borders [16] for experimental data. Dealing with an extensible set of formats in a more generic yet customizable way requires decoupling the software infrastructure from these domain-specific representations. Designing such system is not trivial considering that both experimental and computational data and models each come with their own set of challenges. The sheer size of experimental datasets, particularly those arising from connectomics and imaging, require specific visualization capabilities and optimizations when handling them. Computational models need to be instantiated within an application to let users interact with their state variables and parameters. Different numerical solvers may be required for these models to be simulated, but the user will not necessarily want to be exposed to the complexity of the software solution and low-level libraries involved [17]. In addition, as the biological detail and scale of simulations increase, transparent access to high-performance computing infrastructures [18] will be required. Data and models are also likely to be stored in repositories and databases using disparate technologies, which poses yet another challenge for applications.

To address the challenges posed by heterogeneous data and models, as well as bridging the divide between users with different fields of expertise, we have developed *Geppetto*, an open source, modular middleware platform that can be used to build different neuroscience applications. In order to process diverse types of data and models in a reusable way, the software infrastructure is decoupled from domain data and model specification. This decoupling is achieved through the *Geppetto Model Abstraction*, designed to represent the underlying experimental and computational data and models in a standard way, via reusable modules. Geppetto is also optimized for coping with large amounts of data, through automatic compression and loading on demand, and is able to run simulations on remote supercomputers. To improve accessibility, Geppetto facilitates building novel interfaces by hiding the underlying technologies and by providing prebuilt user-friendly user interface (UI) components. By abstracting and integrating experimental data, computational models and simulators, it is hoped that Geppetto will enable the building of neuroscience applications that can bring together theorists, modellers and experimentalists to formulate and answer increasingly challenging scientific questions related to brain function.

## 2. Methods

**Figure 1.**
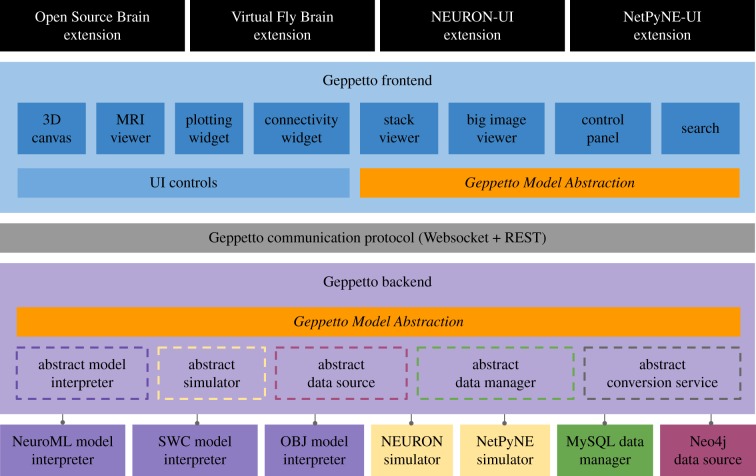
Geppetto architecture. Graphical representation of the components of Geppetto illustrating how the *Geppetto Model Abstraction* (orange blocks) allows backend model and data sources to be accessed by users through browser-based frontend components. Black blocks in the figure are Geppetto *Extensions*, used by applications built on top of the Geppetto platform. The Geppetto frontend (shades of blue) is shown containing a diverse set of visualization components. Communication between the frontend and backend happens via Websockets and a REST-API layer (grey block). The Geppetto backend (light purple block) orchestrates the various services available in a given Geppetto application, including specific *Model Interpreters* (dark purple blocks), external *Simulators* (cream blocks), *Data Managers* (green) and *Data Sources* (pink).

Geppetto is a modular, extensible open-source platform based on a client–server architecture (figure 1) that provides a framework for building neuroscience applications for visualization of data, models and for controlling simulations. The Geppetto backend architecture defines a set of abstract services for which specific implementations can be provided for different domains. The Geppetto frontend provides visualization capabilities that encompass a wide range of what is typically needed for neuroscience data visualization, be it experimental data or data resulting from simulations. The Geppetto frontend is based on a typical modern web stack based on JavaScript and React [19], making use of npm [20] to manage dependencies and webpack [21] to package the code into a browser-ready application.

The *Geppetto Model Abstraction* (figure 1, orange boxes) enables the decoupling of domain-specific modelling formats from the visualization components, by providing a meta-model that can be used to represent them in a declarative way. To this end, it defines a type system based on core concepts from Object-Oriented Programming: *Variables*, *Types* and *Values*. By supporting *Type* inheritance (any Geppetto *Type* can extend another) and composition (Geppetto's *CompositeType* can contain *Variables* of other *Types*), the *Geppetto Model Abstraction* makes it possible to represent hierarchical structures of data and models. Geppetto uses the eclipse modelling framework (EMF) [22] to specify its models' abstractions. The EMF schema is then used to programmatically generate an API for the *Geppetto Model Abstraction* for each one of the supported (user domain) languages [23,24]. Developers can build their own custom *Types* using this *API,* and use them in combination with the ones provided in the *Geppetto Common Library* (e.g. *State Variable*, *Parameter*, etc.). Any model created using the *Geppetto Model Abstraction* takes the name of a *Geppetto Model.* Once a domain-specific model is described in terms of the *Geppetto Model Abstraction* (e.g. by defining a custom *Type*), the entire platform becomes capable of treating its constituent elements appropriately. It is important to note that in Geppetto, *Types* are defined using a domain agnostic meta-model: while an application could, for example, create a *Library* of *Types* that represent computational models, another application might build one whose *Types* represent sets of microscopy images. Inside a *Geppetto Model*, developers can also specify the *Data Source* services used to fetch data from remote repositories, along with the *Queries* available to interrogate them. The *Geppetto Model Abstraction* also defines *ImportTypes* which can hold references to data and models existing on the backend that have not yet been loaded. Sending *ImportTypes* to the client, that will be fully loaded upon a request triggered by the user's actions, is what enables Geppetto to load data on demand (i.e. lazy loading).

The entry point for a Geppetto application is the *Geppetto Project*. Each *Geppetto Project* holds a reference to a single *Geppetto Model* and in addition stores the current state of the application (e.g. which components are open along with their content and position). Every Geppetto application can make use of one or multiple *Geppetto Projects.* For example, in Open Source Brain (OSB) (described below in the Results section), each computational neuroscience model (e.g. cell, network) loaded in from a NeuroML file is mapped to a *Geppetto Model* and contained within a *Geppetto Project*, through which the user will interact with the model.

**Figure 2.**
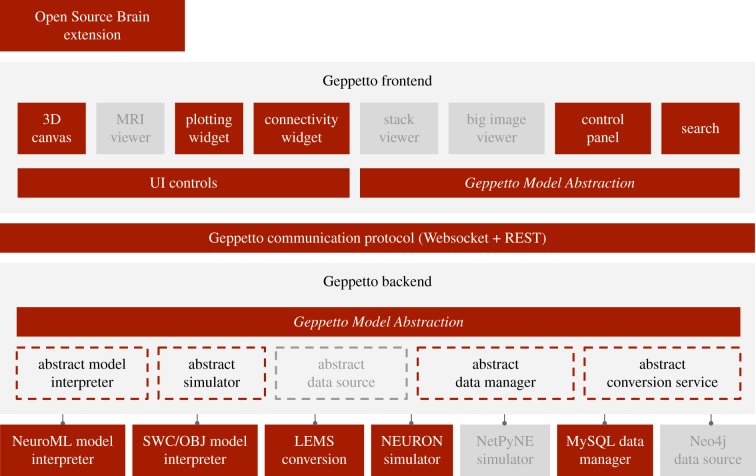
Geppetto OSB configuration. Graphical representation of the components of Geppetto that are used on the OSB application (red). The ones not used are coloured in grey.

**Figure 3.**
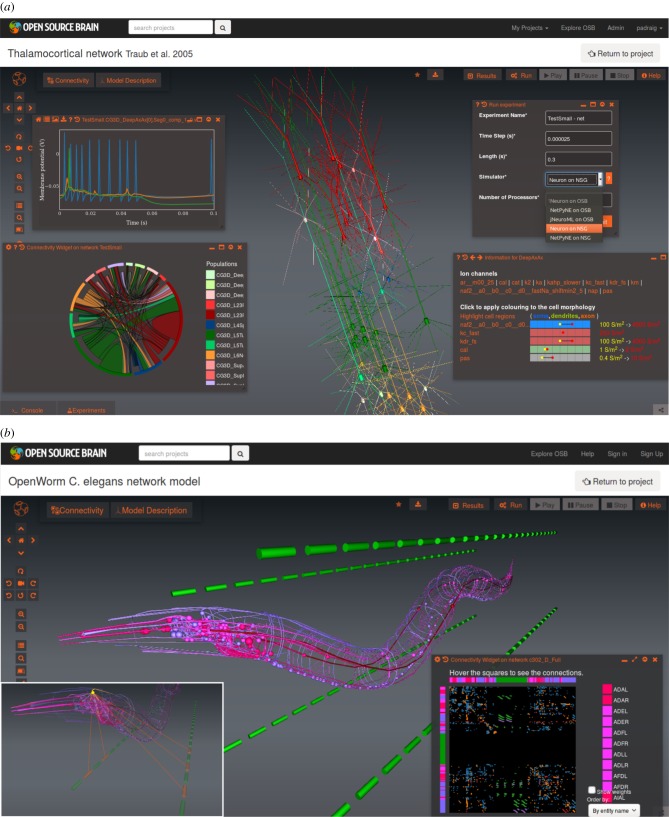
(*a*) Screenshot of a reduced thalamocortical network model [26] on OSB showing analysis and simulation widgets provided by Geppetto and the Geppetto frontend OSB extension. Centre of screen shows 3D rendering of the 12 populations of pyramidal cells and interneurons. Widgets shown are (clockwise from top-left): plot showing recorded membrane potentials from three cells of a previously run experiment; run dialogue for selecting simulators and running experiments; widget showing ion channels and their densities for a single-cell model; chord diagram showing connectivity between populations. (*b*) Visualization of the neuronal network model of *C. elegans* being developed by the OpenWorm project. Centre of screen shows 302 neurons (red: interneurons; pink: sensory; purple: motor neurons) and four quadrants of body wall muscles (green) located away from the body for clarity. Connectivity widget on lower right shows chemical synapses between individual neurons/muscles. Inset on lower left illustrates interactive exploration of network; selecting a single motor neuron (RMED in head) highlights the neurons connected to it, along with five muscles in two of the ventral quadrants.

**Figure 4.**
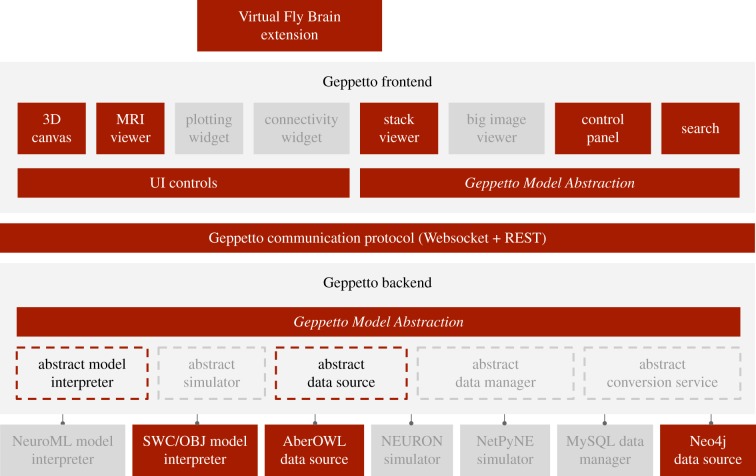
Geppetto VFB configuration. Graphical representation of the components of Geppetto that are used on the VFB application (red). The ones not used are coloured in grey.

The Geppetto backend has a modular architecture that defines multiple service abstractions (figure 1, dashed lines) designed to perform different operations. The specific implementations of these services live in separate modules that can be optionally used by the different applications. For instance, Virtual Fly Brain (VFB) uses the OBJ and SWC [25] *Model Interpreters*, while OSB uses the one for NeuroML (figures 2–4). New modules that implement these service abstractions can be contributed to expand Geppetto's capabilities. The Geppetto backend is responsible for loading in memory *Geppetto Projects* and for delegating the user actions that require server-side operations to the appropriate services, as specified in the *Geppetto Model*. In this regard, the main role of the Geppetto backend is to orchestrate the interactions of all services available in a particular application. A Geppetto backend implementation exists for both Java (the reference, fully featured, one) and Python. Different application servers can be used to host the backend including Virgo [27] for Java and Django [28] or Jupyter [29] for Python. The needs of the specific application will determine the most suitable backend to use, with the Java one currently targeting robust client–server applications aimed at a multi-user deployment (e.g. OSB, VFB) and the Python one also useful for lightweight local deployments aimed at a single user (e.g. NEURON-UI, NetPyNE-UI).

A central abstract service defined in the Geppetto backend is the *Model Interpreter*. Specific *Model Interpreter* implementations are used to let Geppetto essentially ‘understand’ a given format representing concepts in the user's original domain—i.e. they allow building instances of the *Geppetto Model Abstraction* from descriptions in the users' domain language. *Model Interpreters* for popular neuroscience formats such as LEMS [15], NeuroML [14] and NetPyNE [6] are already available.

The abstract *Simulator* service is designed to wrap and control simulators external to Geppetto. The Geppetto backend orchestrates the interactions between *Model Interpreter* and *Simulator* services, so that models can be loaded, converted and simulated as result of user operations. Implementations of the *Simulator* service can wrap simulators as external processes or as remote ones running on external servers (e.g. the Neuroscience Gateway supercomputing facilities [30]). A number of computational neuroscience simulators such as NEURON [3] and NetPyNE [6] have already been wrapped and are available for reuse. Following this architecture, new simulators can be integrated into Geppetto with relative ease.

*Geppetto Data Source* services are similarly implemented extending the provided abstract *Data Sources*, and allow Geppetto to pull in data by querying external systems. Multiple *Data Sources* are configurable in the *Geppetto Model*, making it possible to use Geppetto as a data integration platform. Data fetched from external *Data Sources* can be post-processed to create a representation of the data once again compatible with the *Geppetto Model Abstraction*. VFB [31], a hub for *D. melanogaster* nervous system data built using Geppetto (discussed in detail in the Results section), uses two different implementations of the *Data Source* service, one for Neo4j [32] and one for AberOWL [33], to fetch data from their pre-existing data pipeline. Other *Data Source* services for other types of remote servers could be implemented following these existing examples and the same architecture.

For scenarios where user authentication is required and user data need to be persisted, the *Data Manager* service can be used by developers to configure the backend to enable authentication and database persistence of the *Geppetto Projects* and simulation results.

The Geppetto frontend is responsible for presenting the models and data to the user and for allowing them to interact with the application and its workflows. The Geppetto frontend offers a set of controls and components (figure 1) to build the UI of Geppetto-based applications. While controls (e.g. buttons, dropdowns, dialogs, etc.) are generic and data agnostic building blocks, components are more complex constructs that can be used to display data (e.g. three dimensions (3D), time series, connectivity, MRI, big images, stack, etc.) or to enable specific workflows (e.g. Control Panel, Search, Query, etc.). Components are built using various lower-level JavaScript open-source libraries (e.g. [34–37]) and are designed to integrate with the *Geppetto Model* using a specific API. Any component can be optionally created inside a draggable dialogue window to facilitate data presentation. Components inside these windows are referred to in Geppetto as *Widgets*.

*Geppetto Extensions* let developers decide what controls and components they need for their specific application, control the layout and look and feel and also create additional domain-specific custom components (*Extensions* are represented by the black boxes in figure 1). Geppetto only loads the UI components specified in the *Geppetto Extension* of a given application. A default *Extension* is provided as an example and is accessible via https://live.geppetto.org. By loading the components asynchronously only once the interface needs them, Geppetto optimizes the loading times of the application at start-up.

Upon receiving a *Geppetto Model* from the backend, when loading a given *Geppetto Project*, the frontend will instantiate it. Instantiated Geppetto *Types* are mapped to JavaScript objects (e.g. a population of one cell *Type* would become a JavaScript array containing *Instances* of that *Type*) and augmented with specific *Capabilities* which confer on them the ability to be accessed via a specific API. So, for instance, if a *Model Interpreter* in the backend defined a custom *Type* including a *State Variable,* upon instantiation in the frontend, this would become a JavaScript object with an injected *StateVariableCapability* containing methods specific for state variables, e.g. *getUnit()*, *getInitialValue()*, etc. This has the advantage of giving developers the ability to build UI components that can interact with the *Geppetto Model* in an object-oriented way, and allow all the user operations to be fully scriptable, reproducible and testable (e.g. a UI button designed to plot a state variable would call *Plot.plotData(myStateVariable.getTimeSeries()).* The same principles apply when a custom *Type* defining a cell morphology (*Values* like *Sphere* and *Cylinder* are available to this end in the *Geppetto Model Abstraction*) is sent to the frontend and passed to the *3D Canvas* component using its API for display. Geppetto has the ability to either visualize a single instance of a *Type* (a cell morphology in this example) or an entire population based on it, depending on whether the *Model Interpreter* responsible for the creation of the model instantiated the *Type* only once or multiple times through an *ArrayType*. In some cases, as with the *Stack Viewer* which connects directly to an IIP3D Server [38], it might be preferable for the UI components to read directly a specific format without requiring a mapping to the *Geppetto Model*, which is also permitted by the architecture.

## 3. Results

In this section, we present four examples of neuroscience applications that have been built using Geppetto. Thanks to Geppetto's open-source model, many of the features and components described in the Methods section have evolved in concert with the development of these applications in order to satisfy their requirements. Each of the applications have their own *Extension,* where their custom functionality is specified, and a specific deployment configuration. While the first two, OSB and VFB, use the Java backend and are deployed on public web servers where multiple users can access them simultaneously, the last two, NEURON-UI and NetPyNE-UI, use a Python backend and are designed to be local deployments aimed at a single user, similar to traditional client applications. Geppetto is currently being used to build a total of seven neuroscience applications [31,39–44].

## 4. Open Source Brain

OSB (http://www.opensourcebrain.org) is a platform for visualizing, simulating, disseminating and collaboratively developing standardized, biophysically detailed models of neurons and circuits [45]. OSB contains a range of published neuronal and circuit models from multiple brain regions including the neocortex, cerebellum and hippocampus as well as invertebrate neuron models. Model components (e.g. point neuron or morphologically detailed cell models including membrane conductances, synapses, 3D network structures) are contained in user-created projects, each linked to a public code sharing repository (normally hosted on GitHub) that holds the model source code, specified in NeuroML, a widely used model description format for computational neuroscience [14,15]. OSB provides an integrated browser-based workspace that captures many of the infrastructural demands of projects in computational neuroscience, and allows users to interact with the underlying neuronal models through a graphical interface, without requiring programming knowledge or installing and configuring simulators.

**Figure 5.**
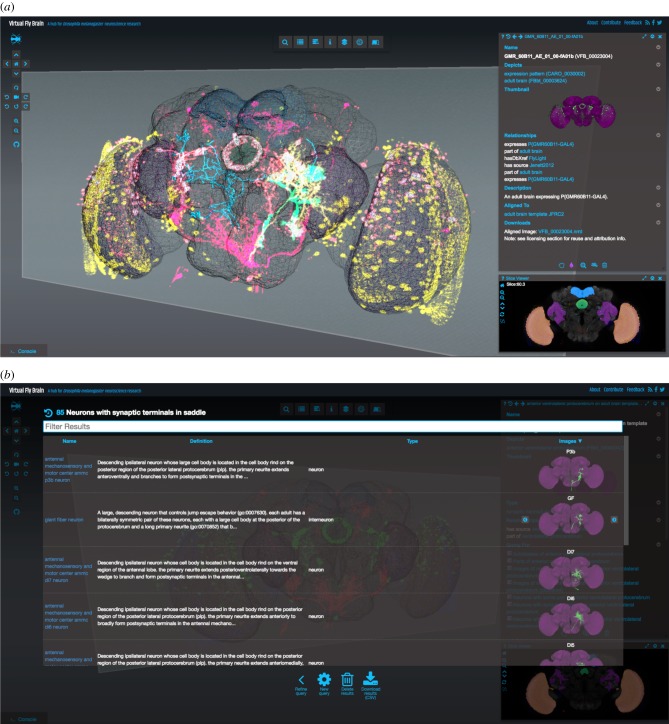
(*a*) VFB main view shows a reference template for the *D. melanogaster* from Janelia Research Campus using the *3D Canvas*. Superposed on the template are various gene expression patterns visualized as point clouds, reconstructed neurons and segmented neuropil regions. At the bottom right corner, the *Stack Viewer* shows a frontal slice through the superposed confocal microscopy images. The Stack Widget is fully synchronized with the 3D Canvas and a moving 3D plane indicates the specific slice currently displayed. On the top of the Stack Widget, a Geppetto viewer is used to display the ontological information associated with the current selection. (*b*) Geppetto's Query component is used to display the results of queries that can be executed from the UI, in this case, the UI shows all the neurons with synaptic terminals in the saddle. By clicking on the thumbnails, the selected neuron is loaded on demand and visualized in the *3D Canvas*, the *Stack Viewer* and the textual definition.

Figure 2 shows how Geppetto is configured for OSB. Many aspects of Geppetto's functionality have been developed to provide the core functionality for OSB. The NeuroML *Model Interpreter* and the LEMS *Conversion* services were contributed to Geppetto to deal with the NeuroML and LEMS formats, reusing previously developed libraries [15]. The NeuroML *Model Interpreter* allows standardized model descriptions to be loaded into the OSB Geppetto deployment, providing automatic 3D visualization of morphologies and internal structure of models, such as state variables and parameters (figure 5*a*) and connectivity within the network (figure 5*b*). Structured metadata in the NeuroML files can be extracted, as well as the underlying mathematical expressions of dynamical components in the model (e.g. kinetics of membrane conductances). These data are made available in an accessible format to the user through a custom *Extension* to the Geppetto frontend.

This OSB custom extension to Geppetto adds shortcuts and menu options for interacting with models, running simulations and visualizing their results. A summary of information extracted from the NeuroML model can be accessed through a ‘Model Description’ widget, which includes links to the source file and original data sources, giving model provenance. This widget also provides easy access to neuronal model-specific functionality, such as plotting rates of activation and inactivation for ion channels and overlaying locations and densities of active conductances on neuronal morphologies (bottom right, figure 5*a*). A shortcut to the *Connectivity Widget* allows the user to see synaptic connectivity of models at a glance: as a chord diagram (bottom left, figure 5*a*), connectivity matrix with weights (bottom right, figure 5*b*), force-directed graph or hive plot. Key parameters present on any given model are thus automatically exposed in a format familiar to neuroscientists.

The simulator agnostic NeuroML format can be converted to simulator-specific formats such as NEURON [3] using a suite of existing converters that implement the Geppetto conversion service interface (figure 2). Geppetto's external simulator abstraction allows OSB to transparently interface with these converters and their associated simulators, allowing models to be simulated through a simple interface. Geppetto can either dispatch simulator jobs to the Neuroscience Gateway [30], a high-performance computing facility or run them on OSB servers. The extension provides assistance for simulation workflows; basic protocols can be defined that create batched experiments with a given range of parameters or the user can record all membrane potentials with a single click. Upon completion, the data generated are sent to the browser for visualization using a Geppetto's plotting widget (top left, figure 5*a*), or recorded membrane potentials or calcium concentrations can be visualized by pseudocolouring the morphologies to show changes over the course of a simulation, and the simulation can be replayed at various speeds. Alternatively, the raw results can be downloaded or automatically uploaded to Dropbox via Geppetto's dropbox interface functionality. Experiments run asynchronously on remote servers, so users do not need to keep their browser open.

The configurable functionality of Geppetto middleware enables OSB to make models accessible, opening them up to critical scientific scrutiny by a wide range of neuroscientists. This supports the process of ongoing model evolution, which is aided by OSB's deep link to GitHub [46], preventing model development from becoming arrested at the point of publication. OSB therefore provides a resource of robust models that can function as best practice examples for model sharing for the neuroscience community.

In addition to this research aspect, OSB also leverages Geppetto's tutorial component to provide interactive computational neuroscience tutorials aimed at students. These tutorials allow users to run virtual experiments and protocols through an easy-to-use web interface, allowing basic concepts in neurophysiology and computational neuroscience to be taught without installing simulators or writing code.

## 5. Virtual Fly Brain

VFB (http://virtualflybrain.org) is a hub for *D. melanogaster* neuroscience research which was born from the need to make the newly standardized fly neuroanatomy available to the public [47–49]. Along with extensive curation of the literature in collaboration with FlyBase [50], VFB v1 allowed users to explore labelled confocal immunofluorescent slices of the adult fly brain across the Internet. The user could step through the brain and identify anatomy by hovering over it. Later this expanded to include expression, transgene and single neuron image data published by multiple laboratories that was aligned to the same template brain enabling any of the 40 000 images to be overlayed. While most researchers were used to viewing slices through the brain, with more single neurons appearing as tiny points in cross-section, interpreting the morphology was increasingly difficult without a 3D representation.

VFB v2 was designed to provide access to all the complex queries and data an expert might require within an interface a novice can easily navigate. Geppetto's existing 3D browser infrastructure atop a flexible modelling framework was used to enable VFB to run complex queries across multiple backend service APIs while maintaining an easy-to-use UI. The *Geppetto Model* has been used to provide abstraction of the specifics of third-party API configuration, query construction and representation into a single simple human-readable file.

Display of the original immunofluorescent confocal gene expression data was implemented in Geppetto as point cloud renderings, while the OBJ *Model Interpreter* was reused to display anatomy regions as surface renderings (figure 5*a*).

**Figure 6.**
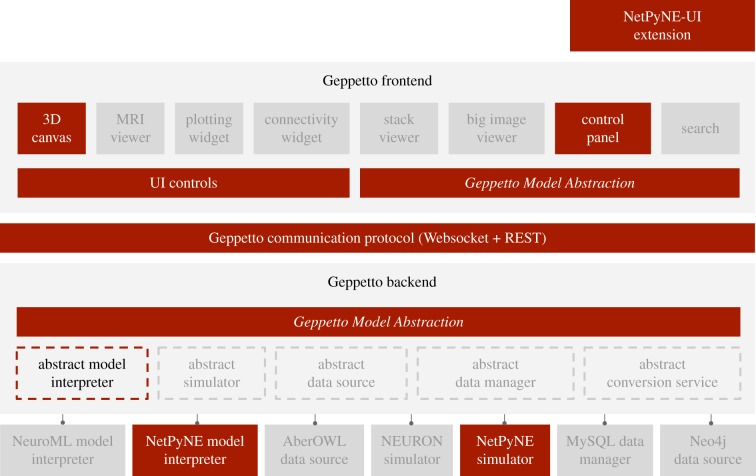
Geppetto NetPyNE-UI configuration. Graphical representation of the components of Geppetto that are used on the NetPyNE-UI application (red). The ones not used are coloured in grey.

A *Model Interpreter* for the SWC format [25] was added to display segmented reconstructions of neuronal morphologies. A *Stack Viewer* was contributed to display 2D confocal microscopy data and synchronized with the pre-existing 3D Canvas component. Geppetto's ability to load data on demand and to optimize the visualization of neurons as tubes or traced lines was essential for VFB to efficiently display larger amounts of imaging data on the screen. The ability to query third-party RESTful APIs through the *Data Source* services allowed VFB to fetch remote data running complex queries (figure 5*b*) involving multiple configurable Data Sources (figure 1). VFB currently pulls data via an ontology reasoner (OWL-ELK [33]) as well as a graph database (Neo4j [32]). Geppetto's Control Panel and Search components were reused and customized within VFB's Geppetto *Extension* to show custom fields and to provide autocompletion search results utilizing a (SOLR [51]) indexing server.

## 6. NEURON-UI and NetPyNE-UI

NEURON is a widely used simulator in the neural multi-scale modelling domain, allowing models to be built that link reaction–diffusion dynamics at the molecular level, to neuronal electrophysiology, up to the large-scale network level [3,6,52,53]. It has thousands of users, a model database [54] with over 600 models, and over 1900 NEURON-based publications. NEURON is being used by major brain research initiatives such as the Human Brain Project and the Allen Institute [18,55]. NEURON includes a native graphical UI for model construction and control, which while fully functional has limited usability and graphical capabilities and is based on deprecated libraries (Interviews) originally developed in the 1980s.

NetPyNE [56] is a high-level Python interface to NEURON that facilitates the development, simulation and analysis of biologically detailed neuronal networks. It provides a unique high-level declarative language designed to facilitate the definition of data-driven multi-scale models (e.g. a concise set of connectivity rules versus millions of explicit cell-to-cell connections). The user can then easily generate NEURON network instances from these specifications, run efficient simulations (including on high-performance parallel computing resources) and exploit the wide array of built-in analysis functions. Its standardized format—compatible with NeuroML—makes it easier to understand, reproduce and reuse models. NetPyNE is being used to develop models of different brain regions—e.g. thalamus, cortex and hippocampus—and phenomena—e.g. neural coding and brain disorders [6,57].

Geppetto has been used to build UIs for both NEURON and NetPyNE. The two applications, designed to be installed and used locally by a single user, have in common an architecture based on the Geppetto interactive Python backend. This backend is implemented as a Jupyter Notebook [29] extension which provides direct communication with the Python kernel. By defining a set of component extensions, Geppetto's interactive Python backend makes it possible to synchronize the data model underlying the UI with a custom Python model. This functionality is at the heart of both NEURON-UI and NetPyNE-UI and means any change made to the Python kernel is immediately reflected in the UI and vice versa.

**Figure 7.**
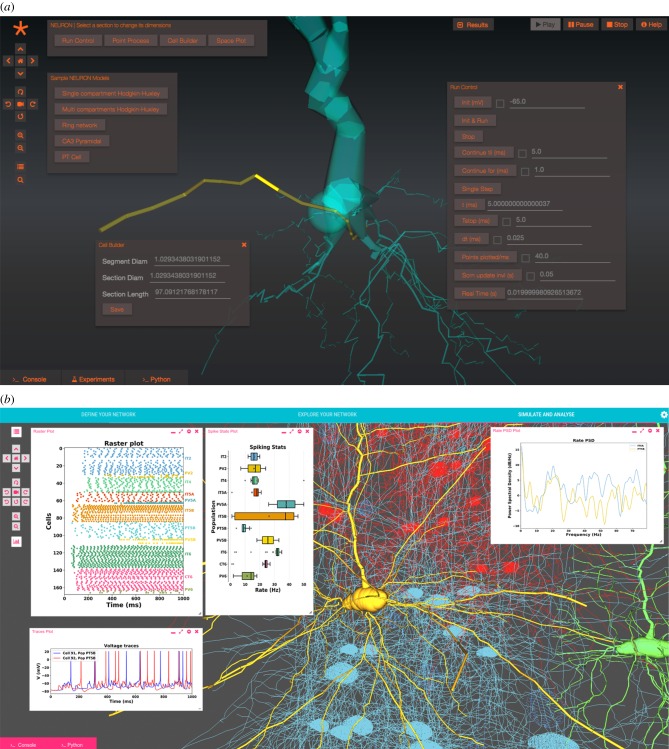
(*a*) Screenshot of NEURON-UI while in edit mode, a simplified cell builder (bottom left) lets the user edit any selected section (in yellow) while the Run control panel (right) is used to control the simulation. (*b*) NetPyNE-UI showing the result of a simulation of a large-scale M1 microcircuit model with widgets showing a raster plot (top left), individual cell membrane potentials (bottom left), population spiking statistics (middle) and the power spectral densities for two populations (right).

Although NEURON-UI and NetPyNE-UI share the same architecture (figure 4 gives an overview of the Geppetto components used in NetPyNE-UI), they differ in certain aspects. In NEURON-UI, the graphical interface is created using a custom Python API meant to mimic NEURON's Interviews-based API. The panels, buttons and text boxes in the UI are therefore created from Python and mapped to Geppetto UI components (figure 7*a*). These components are then connected to the internal Geppetto API to visualize the cells and the networks, run the simulations and plot the results. The idea behind this approach was to retain backward compatibility with the numerous existing NEURON interfaces built with Interviews for various models. Our future aim is to fully map the NEURON API to our NEURON-UI, therefore providing a comprehensive alternative to the traditional UI.

By contrast, in NetPyNE-UI, the UI is defined entirely in JavaScript inside its Geppetto extension. This offers a flexible and intuitive way to create advanced layouts while still enabling each of the elements of the interface to be synchronized with the Python model. The UI splits the workflows in three tabs: network definition, network exploration and network simulation and analysis (figure 7*b*). From the first tab, it is possible to define—or import via Python—the high-level network parameters and rules that will be used for its generation. In the second and third tabs, Geppetto's 3D Canvas is used to visualize the instantiated network. The third tab lets the user simulate the instantiated model (this tab is selected in figure 7*b*). Geppetto allows NetPyNE-UI also to display on the browser a number of plots that are defined in NetPyNE using matplotlib for network analysis and simulation. Both NEURON-UI and NetPyNE-UI can be installed via pip [58] or used inside provided Docker images.

The new Geppetto-based UIs will make NEURON and NetPyNE accessible to a wider range of researchers and students, including those with limited programming experience. This will enable experimentalists to better collaborate with modellers, or to directly reproduce and explore their own experiments via computational simulations.

## 7. Discussion

We have developed Geppetto, an open-source middleware platform for building accessible neuroscience applications. Geppetto facilitates the development of complex applications by providing a well-tested, reusable set of building blocks to integrate diverse neuroscience data, models and simulators. Geppetto provides a modular frontend, where multiple customizable UI components and *Widgets* make it possible to visualize and analyse models and data, as well as a backend capable of connecting to multiple data sources and lower-level, domain-specific descriptions and simulators. This was made possible by designing the *Geppetto Model Abstraction* that can be used to represent a variety of neuroscience domain models, linked to a modular web-based architecture engineered using various open-source libraries. Geppetto has been used as the basis of a number of online and desktop applications in neuroscience: OSB, VFB, NEURON-UI and NetPyNE-UI described here, as well as Patient H.M. [39], WormSim [41] and SciDash [44].

Neuroscience applications are typically developed independently, to address a specific requirement. This leads to considerable redundancy with the same functionality being redesigned and implemented over and over again [59–65]. This approach is only justifiable when the shared set of features is negligible. In this paper, we have shown that even for applications whose requirements were specified independently and had minimal overlap, there can be a significant degree of shared infrastructure. Geppetto proposes an alternative approach by exploiting this fact, allowing neuroscience applications to be built from reusable modules—as illustrated by the overlapping blocks in figures 2–4. This strategy fits naturally into the open-source model—components and modules are more likely to be reusable compared to monoliths—making Geppetto a flexible and extensible solution for multiple applications in neuroscience.

As middleware that factors out commonalities between different domains, Geppetto's modular structure enables a high level of reuse, allowing developers to skip to writing only code specific to their neuroscience application resulting in a considerable saving of time. As with all software platforms, Geppetto has its own learning curve required for developers to understand its architecture and become familiar with its components. While at first this initial investment might be seen as a complication compared to the apparent ease of starting from a blank slate, developers associated with the applications described above, with no previous experience on Geppetto, have found it only takes from one to four weeks^1^ to become productive. This time investment is outweighed by the subsequent savings made in avoiding common pitfalls, replicating solutions to common problems and rewriting entire software components and workflows. There is also a significant advantage in interacting with the active community of Geppetto developers, who can assist with any queries. The net time saving compared to an approach that starts from scratch is difficult to estimate but is likely to range from six months to five years^2^ depending on the targeted scope—the more the features required that overlap with Geppetto's the bigger the savings—and on the size and experience of the team of developers involved. Moreover, extensive sharing of modules between applications results in them being thoroughly tested [66], while having a shared infrastructure that undergoes regular release cycles ensures maintenance is less burdensome for each specific application. Furthermore, the distributed nature of the Geppetto code base and the fact that updates are made independently of any specific project ultimately increases the longevity of any application built with this platform.

The diversity of applications that have been built so far with Geppetto illustrates the flexibility of its model abstraction capabilities, which can encompass different domains, data and scientific modelling formalisms. Also, as the platform keeps evolving, new solutions added for a specific application become immediately available to all the other applications. Examples of this include many of the features contributed by OSB being reused by multiple applications (e.g. *Control Panel*, *Search Bar* or the *Experiments table*); the SWC [25] *Model Interpreter* contributed by VFB, which is reused in OSB; and the *3D Canvas*, originally built for the first deployment of the platform and reused by every other application to date. Geppetto combines a model-driven design with a service-oriented architecture to enable reuse across multiple applications. Its modularity, a centrepiece of both the backend and the frontend, is obtained by engineering together a unique set of technologies [19,21,22,67,68] to provide novel functionality. By allowing different neuroscience applications to use the same technologies, Geppetto provides well-tested solutions that bring closer together otherwise disjoint research groups—both computational and experimental, thereby fostering collaboration.

**Figure 8.**
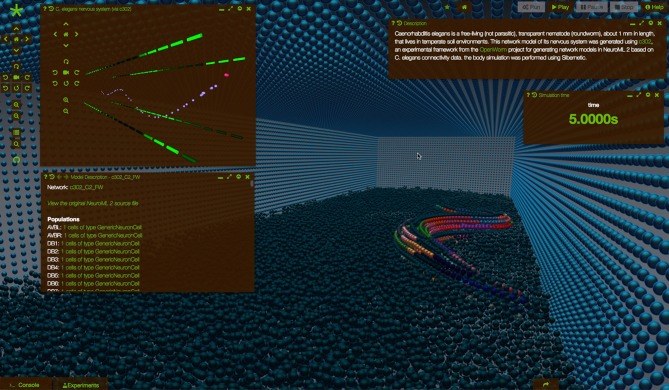
Prototype of the integration between a nervous system model (top-left widget) and a fluid mechanics–based simulation of a worm body (background *3D Canvas*) within Geppetto, currently under development. The mechanical model of the body of the worm, which includes musculature, is shown immersed in a simulated fluid environment. Both worm body and fluid are made of particles. Different colours on the worm body highlight different groups of particles (e.g. elastic particles for each of the worm muscles, liquid particles for the surrounding fluid, etc.). All around the fluid and the worm is the experiment bounding box, made of an impermeable layer of particles. The calcium concentrations in the muscles (four rows separated from the main body cells in top-left widget) simulated by the model are translated into activation signals for the muscles cells in Sibernetic ultimately driving the locomotion of the worm.

The Geppetto applications described in the Results section are in active development. Some of the planned and ongoing projects include: extending OSB to bring together models and the experimental data used to build and test them, by adding standardized data interpreters (e.g. v. 2 of the Neurodata Without Borders format); extending VFB to cover all stages/regions of the fly CNS, incorporating synapse level connectomics data with the extensive light level image and literature knowledge; releasing a new version of WormSim, currently being developed within the OpenWorm project [1] that will integrate the Sibernetic [69] fluid dynamics simulator (see [70]) with the NeuroML-based nervous system model (see [71]). The latter will be the first instance of a Geppetto application providing a non-computational neuroscience-specific numerical engine, used for fluid dynamics simulations (figure 8).

Thanks to its open, modular, web-based architecture, Geppetto ultimately enables the engineering of a new breed of neuroscience applications that can be used in a collaborative way by theoreticians, modellers and experimentalists to formulate new scientific hypotheses, build and validate new models, and help gain insights into the most pressing questions in neuroscience.

## Endnotes

1 Depending on the background and level of experience of the developer.

2 Estimate based on the actual time that was spent designing and implementing various reusable components, e.g. 3D Canvas 6 months, MRI Viewer 3 months, Plotting widget 6 months, Connectivity Widget 5 months, Stack Viewer 6 months, Control Panel 3 months, *Geppetto Model Abstraction* 9 months, etc. Building of the infrastructure in its current form took 3 years. All these figures consider 1 Senior Development Engineer FTE.

## Data accessibility

Geppetto is open source (http://git.geppetto.org) and released under the MIT licence. Documentation is available at http://docs.geppetto.org. A live demo application to showcase the latest release of Geppetto (0.4.0 at the time of writing, new versions are released monthly) is available at http://live.geppetto.org. Docker images are available for Geppetto at http://docker.geppetto.org, which simplify creation of a local instance of the application with all required libraries preconfigured. Integration tests for the full stack and for the UI are available for all the main features. These tests are automatically executed after every commit.

## References

[1] Szigeti B et al. 2014 OpenWorm: an open-science approach to modeling *Caenorhabditis elegans*. Front. Comput. Neurosci. 8, 137 (10.3389/fncom.2014.00137) 25404913 PMC4217485

[2] Lamaze A, Öztürk-Çolak A, Fischer R, Peschel N, Koh K, Jepson JEC 2017 Regulation of sleep plasticity by a thermo-sensitive circuit in *Drosophila*. Sci. Rep. 7, 40304 (10.1038/srep40304) 28084307 PMC5233985

[3] Carnevale NT, Hines ML 2006 The NEURON book. Cambridge, UK: Cambridge University Press.

[4] Gewaltig M-O, Marc-Oliver G, Markus D 2007 NEST (NEural Simulation Tool). Scholarpedia J. 2, 1430.

[5] Goodman DFM, Brette R 2009 The brian simulator. Front. Neurosci. 3, 192–197. (10.3389/neuro.01.026.2009) 20011141 PMC2751620

[6] Lytton WW, Seidenstein AH, Dura-Bernal S, McDougal RA, Schürmann F, Hines ML 2016 Simulation neurotechnologies for advancing brain research: parallelizing large networks in NEURON. Neural Comput. 28, 2063–2090. (10.1162/NECO_a_00876) 27557104 PMC5295685

[7] Somogyi ET, Bouteiller J-M, Glazier JA, König M, Medley JK, Swat MH, Sauro HM 2015 libRoadRunner: a high performance SBML simulation and analysis library. Bioinformatics 31, 3315–3321. (10.1093/bioinformatics/btv363) 26085503 PMC4607739

[8] Cannon RC et al. 2007 Interoperability of neuroscience modeling software: current status and future directions. Neuroinformatics 5, 127–138. (10.1007/s12021-007-0004-5) 17873374 PMC2658651

[9] Hines ML, Davison AP, Muller E 2009 NEURON and Python. Front. Neuroinform. 3, 1 (10.3389/neuro.11.001.2009) 19198661 PMC2636686

[10] Bednar JA 2009 Topographica: building and analyzing map-level simulations from Python, C/C++, MATLAB, NEST, or NEURON components. Front. Neuroinform. 3, 8 (10.3389/neuro.11.008.2009) 19352443 PMC2666198

[11] Muller E, Eilif M, Bednar JA, Markus D, Marc-Oliver G, Michael H, Davison AP 2015 Python in neuroscience. Front. Neuroinform. 9, 11 (10.3389/fninf.2015.00011) 25926788 PMC4396193

[12] Dura-Bernal S, Zhou X, Neymotin SA, Przekwas A, Francis JT, Lytton W 2015 Cortical spiking network interfaced with virtual musculoskeletal arm and robotic arm. Front. Neurorobot. 9, 13 (10.3389/fnbot.2015.00013) 26635598 PMC4658435

[13] Bennett KH, Rajlich VT 2000 Software maintenance and evolution: a roadmap In Proceedings of the Conference on the Future of Software Engineering, pp. 73–87. ACM.

[14] Gleeson P et al. 2010 NeuroML: a language for describing data driven models of neurons and networks with a high degree of biological detail. PLoS Comput. Biol. 6, e1000815 (10.1371/journal.pcbi.1000815) 20585541 PMC2887454

[15] Cannon RC, Gleeson P, Crook S, Ganapathy G, Marin B, Piasini E, Silver RA 2014 LEMS: a language for expressing complex biological models in concise and hierarchical form and its use in underpinning NeuroML 2. Front. Neuroinform. 8, 79 (10.3389/fninf.2014.00079) 25309419 PMC4174883

[16] Teeters JL et al. 2015 Neurodata without borders: creating a common data format for neurophysiology. Neuron 88, 629–634. (10.1016/j.neuron.2015.10.025) 26590340

[17] Djurfeldt M et al. 2010 Run-time interoperability between neuronal network simulators based on the MUSIC framework. Neuroinformatics 8, 43–60. (10.1007/s12021-010-9064-z) 20195795 PMC2846392

[18] Markram H et al. 2015 Reconstruction and simulation of neocortical microcircuitry. Cell 163, 456–492. (10.1016/j.cell.2015.09.029) 26451489

[19] *React—a JavaScript library for building user interfaces* See https://reactjs.org/ (accessed on 8 March 2018).

[20] *npm* See https://www.npmjs.com/ (accessed on 28 February 2018).

[21] Xilinx I 2008 Webpack. PlanAHead User Guide 5, 125–156.

[22] Gronback RC 2009 Eclipse modeling project. Boston, MA, USA: Addison-Wesley Professional.

[23] *org.geppetto.model*. See https://github.com/openworm/org.geppetto.model (accessed on 24 February 2018).

[24] *pygeppetto* See https://github.com/openworm/pygeppetto.

[25] *SWC—specification*. See http://www.neuronland.org/NLMorphologyConverter/MorphologyFormats/SWC/Spec.html (accessed on 24 February 2018).

[26] Traub RD et al. 2005 Single-column thalamocortical network model exhibiting gamma oscillations, sleep spindles, and epileptogenic bursts. J. Neurophysiol. 93, 2194–2232. (10.1152/jn.00983.2004) 15525801

[27] *Eclipse Virgo* See http://www.eclipse.org/virgo/ (accessed on 25 February 2018).

[28] Django D 2007 Django The Web framework. Culver City, CA: Django Web Foundation.

[29] IOS Press Ebooks—Jupyter Notebooks—a publishing format for reproducible computational workflows, 2016 See http://ebooks.iospress.nl/publication/42900 (accessed on 25 February 2018).

[30] Sivagnanam S, Majumdar A, Yoshimoto K 2013 Introducing the neuroscience gateway.

[31] *VFB—Virtual Fly Brain, a hub for* Drosophila melanogaster *neuroscience research*. See http://v2.virtualflybrain.org (accessed on 24 February 2018).

[32] Developers N. 2012 Neo4 J *Graph NoSQL Database [online]*.

[33] Slater L, Gkoutos GV, Schofield PN, Hoehndorf R 2015 AberOWL: an ontology portal with OWL EL reasoning. In *mons Database*, p. 127.

[34] *three.js–Javascript 3D library*. See https://threejs.org/ (accessed on 10 March 2018).

[35] *Modern visualization for the data era.* See https://plot.ly/ (accessed on 10 March 2018).

[36] ami. Github, in press. See https://github.com/FNNDSC/ami.

[37] Bostock M, Ogievetsky V, Heer J 2011 D**^3^**: data-driven documents. IEEE Trans. Vis. Comput. Graph. 17, 2301–2309. (10.1109/TVCG.2011.185) 22034350

[38] Husz ZL, Burton N, Hill B, Milyaev N, Baldock RA 2012 Web tools for large-scale 3D biological images and atlases. BMC Bioinformatics 13. 10.1186/1471-2105-13-122 PMC3412715 22676296

[39] *The brain observatory*. See https://www.thebrainobservatory.org/project-hm/ (accessed on 24 February 2018).

[40] *Open Source Brain.* See http://opensourcebrain.org (accessed on 24 February 2018).

[41] *WormSim*. See http://wormsim.org (accessed on 24 February 2018).

[42] *NetPyNE-UI*. See https://github.com/MetaCell/NetPyNE-UI.

[43] *NEURON-UI*. See https://github.com/MetaCell/NEURON-UI.

[44] *SciDash*. See http://scidash.org (accessed on 24 February 2018).

[45] Gleeson P et al. 2018 *Open Source Brain*: a collaborative resource for visualizing, analyzing, simulating and developing standardized models of neurons and circuits. *bioRxiv*. 229484 (10.1101/229484)

[46] *GitHub*. See https://github.com.

[47] Armstrong JD, van Hemert JI 2009 Towards a virtual fly brain. Philos. Trans. R. Soc. A 367, 2387–2397. (10.1098/rsta.2008.0308) 19414461

[48] Costa M, Reeve S, Grumbling G, Osumi-Sutherland D 2013 The Drosophila anatomy ontology. J. Biomed. Semantics 4, 32 (10.1186/2041-1480-4-32) 24139062 PMC4015547

[49] Ito K et al. 2014 A systematic nomenclature for the insect brain. Neuron 81, 755–765. (10.1016/j.neuron.2013.12.017) 24559671

[50] Tweedie S et al. 2008 FlyBase: enhancing Drosophila gene ontology annotations. Nucleic Acids Res. 37, D555–D559. (10.1093/nar/gkn788) 18948289 PMC2686450

[51] Smiley D, Pugh E, Parisa K, Mitchell M 2015 Apache solr enterprise search server, 3rd edn Birmingham, UK: Packt Publishing Ltd.

[52] Tikidji-Hamburyan RA, Narayana V, Bozkus Z, El-Ghazawi TA 2017 Software for brain network simulations: a comparative study. Front. Neuroinform. 11, 46 (10.3389/fninf.2017.00046) 28775687 PMC5517781

[53] McDougal R, Hines M, Lytton W 2013 Reaction-diffusion in the NEURON simulator. Front. Neuroinform. 7, 28 (10.3389/fninf.2013.00028) 24298253 PMC3828620

[54] ModelDB: Home, in press. See https://senselab.med.yale.edu/modeldb/ (accessed on 28 February 2018).

[55] Hawrylycz M *et al.* 2016 Inferring cortical function in the mouse visual system through large-scale systems neuroscience. Proc. Natl Acad. Sci. USA 113, 7337–7344. (10.1073/pnas.1512901113) 27382147 PMC4941493

[56] Dura-Bernal S, Suter B, Neymotin S, Kerr C, Quintana A, Gleeson P, Shepherd GMG, Lytton WW 2016 NetPyNE: a Python package for NEURON to facilitate development and parallel simulation of biological neuronal networks In *Computational Neuroscience (CNS)*.

[57] Dura-Bernal S, Neymotin SA, Suter BA, Shepherd G. 2018 Long-range inputs and H-current regulate different modes of operation in a multiscale model of mouse M1 microcircuits. *bioRxiv*.

[58] pip Python Package Index, in press. See https://pypi.python.org/pypi/pip (accessed on 1 March 2018).

[59] Mark E 2011 The Whole Brain Catalog: platform for neuroscientific data integration. Front. Neuroinform. 5, 137 (10.3389/conf.fninf.2011.08.00137)

[60] Ukani NH et al. 2016 The Fruit Fly Brain Observatory: from structure to function. *bioRxiv*. 092288 (10.1101/092288)

[61] Sunkin SM, Ng L, Lau C, Dolbeare T, Gilbert TL, Thompson CL, Hawrylycz M, Dang C 2013 Allen Brain Atlas: an integrated spatio-temporal portal for exploring the central nervous system. Nucleic Acids Res. 41, D996–D1008. (10.1093/nar/gks1042) 23193282 PMC3531093

[62] Cessac B, Kornprobst P, Kraria S, Nasser H, Pamplona D, Portelli G, Viéville T 2017 PRANAS: a new platform for retinal analysis and simulation. Front. Neuroinform. 11, 49 (10.3389/fninf.2017.00049) 28919854 PMC5585572

[63] Leon PS, Knock SA, Woodman MM, Domide L, Mersmann J, McIntosh AR, Jirsa V 2013 The Virtual Brain: a simulator of primate brain network dynamics. Front. Neuroinform. 7, 10. 23781198 10.3389/fninf.2013.00010 PMC3678125

[64] Brain Simulation Platform, in press. See https://www.humanbrainproject.eu/en/brain-simulation/brain-simulation-platform/ (accessed on 8 March 2018).

[65] Tomita M et al. 1999 E-CELL: software environment for whole-cell simulation. Bioinformatics 15, 72–84. (10.1093/bioinformatics/15.1.72) 10068694

[66] Wikipedia contributors. 2018 Linus's Law. *Wikipedia, The Free Encyclopedia*. See https://en.wikipedia.org/w/index.php?title=Linus%27s_Law&oldid=818874010 (accessed on 8 March 2018).

[67] Alliance O 2003 Osgi service platform, release 3. Amsterdam, Netherlands: IOS Press, Inc.

[68] Spring, in press. See https://spring.io/ (accessed on 11 March 2018).

[69] Palyanov A, Khayrulin S, Larson SD 2016 Application of smoothed particle hydrodynamics to modeling mechanisms of biological tissue. Adv. Eng. Softw. 98, 1–11. (10.1016/j.advengsoft.2016.03.002)

[70] Palyanov A, Khayrulin S, Larson SD 2018 Three-dimensional simulation of the *Caenorhabditis elegans* body and muscle cells in liquid and gel environments for behavioural analysis. Phil. Trans. R. Soc. B 373, 20170376 (10.1098/rstb.2017.0376) 30201840 PMC6158221

[71] Gleeson P, Lung D, Grosu R, Hasani R, Larson SD 2018 c302: a multiscale framework for modelling the nervous system of *Caenorhabditis elegans*. Phil. Trans. R. Soc. B 373, 20170379 (10.1098/rstb.2017.0379) 30201842 PMC6158223

